# Metabolomics analysis of follicular fluid coupled with oocyte aspiration reveals importance of glucocorticoids in primate periovulatory follicle competency

**DOI:** 10.1038/s41598-021-85704-6

**Published:** 2021-03-22

**Authors:** Sweta Ravisankar, Carol B. Hanna, Kelsey E. Brooks, Melinda J. Murphy, Nash Redmayne, Junghyun Ryu, Jason M. Kinchen, Shawn L. Chavez, Jon D. Hennebold

**Affiliations:** 1 Department of Cell, Developmental and Cancer Biology, Graduate Program in Molecular & Cellular Biosciences, Oregon Health & Science University School of Medicine, Portland, OR USA; 2 Division of Reproductive and Developmental Sciences, Oregon National Primate Research Center, Beaverton, OR USA; 3 Department of Obstetrics and Gynecology, Oregon Health & Science University School of Medicine, Portland, OR USA; 4 Metabolon, Inc., Morrisville, NC 27560 USA; 5 Department of Molecular and Medical Genetics, Oregon Health & Science University School of Medicine, Portland, OR USA

**Keywords:** Metabolomics, Embryogenesis, Infertility

## Abstract

Gonadotropin administration during infertility treatment stimulates the growth and development of multiple ovarian follicles, yielding heterogeneous oocytes with variable capacity for fertilization, cleavage, and blastocyst formation. To determine how the intrafollicular environment affects oocyte competency, 74 individual rhesus macaque follicles were aspirated and the corresponding oocytes classified as failed to cleave, cleaved but arrested prior to blastulation, or those that formed blastocysts following in vitro fertilization. Metabolomics analysis of the follicular fluid (FF) identified 60 unique metabolites that were significantly different between embryo classifications, of which a notable increase in the intrafollicular ratio of cortisol to cortisone was observed in the blastocyst group. Immunolocalization of the glucocorticoid receptor (GR, NR3C1) revealed translocation from the cytoplasm to nucleus with oocyte maturation in vitro and, correlation to intrafollicular expression of the 11-hydroxy steroid dehydrogenases that interconvert these glucocorticoids was detected upon an ovulatory stimulus in vivo. While NR3C1 knockdown in oocytes had no effect on their maturation or fertilization, expansion of the associated cumulus granulosa cells was inhibited. Our findings indicate an important role for NR3C1 in the regulation of follicular processes via paracrine signaling. Further studies are required to define the means through which the FF cortisol:cortisone ratio determines oocyte competency.

## Introduction

Since the advent of human in vitro fertilization (IVF) in 1978, the resultant live-birth rates have remained relatively constant at ~ 35%, although the demand has been steadily growing (cdc.gov/art). IVF requires the use of controlled ovarian stimulation (COS) to obtain sufficient quantities of eggs by administering pharmacological levels of exogeneous hormones to stimulate the development of multiple follicles. However, the forced growth and development of multiple follicles causes a concomitant increase in heterogeneity in terms of granulosa cell and oocyte competency because the processes leading to either atresia or selection of a single dominant follicle during the natural menstrual cycle are overridden^1–4^. Such follicular heterogeneity manifests in a significant proportion of embryos arresting prior to forming blastocysts, which likely contributes to low birth rates following IVF. Thus, it is essential to accurately and non-invasively identify those oocytes that possess the greatest potential to fertilize, undergo cleavage divisions, and reach the blastocyst stage early in development to improve IVF success and pregnancy outcomes.

Ovarian follicle maturation leading to the ovulation of a fertilizable oocyte involves complex intracellular interactions between the oocyte itself, surrounding somatic cells, and the follicular fluid (FF) that is derived from both circulating and intrafollicular derived factors^5–14^. The mid-cycle surge of luteinizing hormone (LH), the master regulator of ovulation, triggers direct LH/chorionic gonadotropin receptor signaling and the production of autocrine or paracrine factors responsible for the resumption of meiosis and the transition from an immature germinal vesicle (GV) to a mature metaphase II (MII) oocyte^15–17^. This includes the synthesis and secretion of certain fatty acid-derived and steroid-based hormones, cytokines, chemokines, as well as growth factors by the cells comprising the periovulatory follicle, which in turn, regulate oocyte maturation and fertilization potential. Due to species-specific differences in follicle size, growth rates, and the processes involved in selection, there is limited information from an animal model that is more similar to humans in terms of ovarian physiology for defining the optimal follicular microenvironment that can be extrapolated to women^18^.

Although IVF success depends on multiple factors, much of the research has focused on the correlation between IVF outcomes and somewhat subjective assessments of embryo quality for selection and transfer^19,20^. Non-invasive technologies, including time-lapse imaging of embryo development and analysis of spent culture media, as well as more invasive techniques such as pre-implantation genetic screening (PGS), have been implemented into clinical practice for this purpose, but not without limitations and controversy^21–28^. More recently, studies have concentrated on the cellular and molecular processes occurring in the ovarian follicle at the time of aspiration and oocyte collection. Reports detailing the use of genomic, transcriptomic, and metabolomics approaches to non-invasively assess the intrafollicular microenvironment have begun to emerge and have yielded insight into oocyte competency within the naturally selected follicle and following environmental or pathological insults^29–34^. Most of the metabolomics studies, however, used methods that were only capable of identifying a select number of metabolites involved in known cellular processes. Because the metabolome constitutes inputs from genomic, transcriptomic, and proteomic processes, it is essential that metabolomics analyses be as thorough as possible for the discovery of important, but yet to be characterized, metabolic pathways. Thus, non-targeted mass spectrometry of FF obtained from individual follicles from rhesus macaque females undergoing COS was performed here to define the follicular metabolome relative to the developmental outcome of each resident oocyte following fertilization. Our findings demonstrate that an unbiased metabolomics analysis of FF provides a comprehensive assessment of the intrafollicular signaling pathways and identifies important downstream mediators in follicles containing oocytes with the greatest potential of yielding a normal term pregnancy.

## Materials and methods

### Collection of rhesus macaque oocytes, somatic cells, and FF

All protocols involving animals were approved by the ONPRC Institutional Animal Care and Use Committee and conducted in accordance with the National Institutes of Health Guidelines for the Care and Use of Laboratory Animals, and Animal Research: Reporting of In Vivo Experiments guidelines. The housing and general care of rhesus macaques (*Macaca mulatta*) was previously described^35^.

Female rhesus macaques (N = 17) of reproductive age [~ 7–8 years (yrs) old] underwent COS protocols as previously described over a period of 2 yrs to stimulate the development of multiple ovarian follicles^1^. Female rhesus macaques were anesthetized for laparoscopic follicular aspirations 36 h (h) after the administration of human chorionic gonadotropin (hCG) to induce events necessary for the re-initiation of meiosis. Individual ovarian follicles (N = 10 per ovary) were manually aspirated and collected into separate sterile 1.5 ml Eppendorf tubes with a low dead-space 3 ml syringe with a 22-gauge X 1.5-inch needle (Ulticare, UltaMed Inc., Excelsior, MN). The tubes were centrifuged at 1000*g* for 30 s (sec) at room temperature to separate the FF from the cumulus-oocyte complex (COC) and the granulosa cells (GCs). COCs were then examined for presence of an oocyte under a stereomicroscope by dilution with Tyrode's albumin lactate pyruvate (TALP)-HEPES media with 0.3% bovine serum albumin (BSA; Sigma-Aldrich, St. Louis, MO) and transferred to a pre-equilibrated IVF dish containing 100 μl drops of (0.3% BSA and 0.006% sodium pyruvate) covered by mineral oil (Sage, Trumbull, CT). Each oocyte from an individual aspirate was placed in a separate drop. The FF and the cumulus cells (CCs) were kept in separate tubes, flash frozen in liquid nitrogen, and stored at − 80 °C until future use.

### IVF and assessment of pre-implantation development

Fresh semen from adult male rhesus monkeys of reproductive age (6–9 years) was collected according to established ONPRC protocols^36^ and used for IVF at a final concentration of 2 × 10^6^ sperm/ml in Tyrode's albumin lactate pyruvate (TALP)-Complete media (0.3% BSA and 0.006% sodium pyruvate). Semen was collected only from males that were proven breeders. IVF was performed the evening of the collection as previously described^36^. IVF dishes were incubated at 5% CO_2_ and 37 °C overnight and fertilized oocytes separated from excess sperm the next morning by micropipetting. The zygotes (identified by two pronuclei and/or two polar bodies) were cultured in separate wells containing 100 μl of one-step commercial media supplemented with 10% serum protein (LifeGlobal, Guildford, CT) under mineral oil at 37 °C with 6% CO_2_, 5% O_2_. Embryo development was individually tracked. The culture medium was changed on day 3 post-IVF and the embryos were allowed to continue developing until they arrested or reached the blastocyst stage for a maximum of 8 days post-IVF. Arrested (pre-blastocyst stage) embryos and blastocyst development outcomes were recorded. Based on their fertilization and developmental outcome, embryos were categorized as uncleaved (MII oocytes that did not fertilize or those that appeared to fertilize, but did not cleave), cleavage stage (those that cleaved, but ceased dividing prior to forming a blastocyst), or blastocyst (those that progressed to form a blastocyst).

### FF sample preparation for metabolomics analysis

A total of 255 oocytes were isolated from individual follicles, fertilized via conventional IVF and allowed to undergo pre-implantation development. Out of these, a total 74 individual FF samples (33 from year 1 and 41 from year 2) were categorized based on the corresponding oocyte’s development into the above embryo groups, were shipped in separate batches on dry ice to Metabolon Inc. (Durham, NC, U.S.A.). These FF samples were chosen based on a required FF volume of greater than or equal to 40 μl, which was dictated by the sensitivity of the mass spectrometry platform used for the metabolomics assessment. Further processing and metabolomics analyses were conducted using the automated MicroLab STAR system from Hamilton Company. Recovery standards were added prior to the first step in the extraction process to assess variability and verify performance of extraction and instrumentation. To remove protein and recover chemically diverse metabolites, dissociated small molecules bound to protein or trapped in the precipitated protein matrix, were precipitated in methanol with vigorous shaking (Glen Mills GenoGrinder 2000) for 2 min (min) followed by centrifugation. The resulting extract was split and used by separate reverse phase (RP)/ultrahigh performance liquid chromatography-tandem mass spectroscopy (UPLC-MS/MS) methods with negative ion mode electrospray ionization (ESI), one for analysis by hydrophilic interaction chromatography (HILIC)/UPLC-MS/MS with negative ion mode ESI, and either one or two aliquots for analysis with UPLC-MS/MS with positive mode ESI (depending on year of acquisition). For the first run, a gas chromatography (GC)/MS instrument was also used—samples were dried under vacuum for a minimum of 18 h prior to being derivatized under dried nitrogen using bistrimethyl-silyltrifluoroacetamide. Derivatized samples were separated on a 5% diphenyl/95% dimethyl polysiloxane fused silica column (20 m × 0.18 mm ID; 0.18 um film thickness) with helium as carrier gas and a temperature ramp from 60° to 340 °C in a 17.5 min period. Samples were analyzed on a Thermo-Finnigan Trace DSQ fast-scanning single-quadrupole mass spectrometer using electron impact ionization (EI) and operated at unit mass resolving power. The scan range was from 50 to 750 m/z.

### Quality assessment of metabolomics analysis

Several types of controls were analyzed in concert with the experimental samples, including: 1) a pooled matrix sample generated by taking a small volume of each experimental sample that served as a technical replicate throughout the analysis, 2) extracted water samples that served as process blanks, and 3) a cocktail of QC standards that were carefully chosen not to interfere with the measurement of endogenous compounds and spiked into every analyzed sample, allowing instrument performance monitoring and chromatographic alignment. Experimental samples were randomized across the platform run with QC samples spaced evenly among the injections, as outlined in Supplementary Fig. S1.

### Ultrahigh performance liquid chromatography-tandem mass spectroscopy

Molecules were separated using a Waters ACQUITY UPLC system and identified using a Thermo Scientific Q Exactive Hybrid Quadrupole-Orbitrap mass spectrometer interfaced with a heated electrospray ionization (HESI-II), operating at 35,000 mass resolution. The sample extract was dried and then reconstituted in solvents compatible to each of the four different methods. Each reconstitution solvent contained a series of standards at fixed concentrations to ensure injection and chromatographic consistency. Aliquots were analyzed using acidic positive ion conditions chromatographically optimized for hydrophilic compounds, hydrophobic compounds, acidic positive ion conditions as well as hydrophobic compounds and basic negative ions optimized conditions using a separate dedicated C18 column with negative ionization. The analysis alternated between MS and data-dependent MS^n^ scans using dynamic exclusion and the scan range varied slightly between methods, covering 70–1000 m/z.

### Metabolite identification, quantification, and visualization

Raw data was extracted, peak-identified, and QC processed as previously described^37^. Compounds were identified by comparison to library entries of purified standards or recurrent unknown entities. Biochemical identification was based on three additional criteria: retention index (RI) within a narrow RI window of the proposed identification, accurate mass match to the library +/−10 ppm, and the MS/MS forward and reverse scores between the experimental data and authentic standards. The MS/MS scores were based on a comparison of the ions present in the experimental spectrum to the ions present in the library spectrum. While there may be similarities between molecules according to one of these factors, the use of all three data points was utilized to distinguish biochemicals. More than 3,300 commercially available purified standard compounds were acquired and registered into LIMS for analysis on all platforms and determination of their analytical characteristics. Additional mass spectral entries were created for structurally unnamed biochemicals, which have been identified by virtue of their recurrent nature (both chromatographic and mass spectral). Library matches for each compound were checked for each sample and corrected if necessary. Peaks were quantified using area-under-the-curve. For studies spanning multiple days, a normalization step was performed to correct variation resulting from inter-day instrument differences. For purposes of data visualization, values were normalized in terms of raw area counts.

### Measurement of cortisol and cortisone concentrations in FF by LC–MS

FF was retrieved from one follicle in each ovary of female rhesus macaques undergoing a COS protocol without (0 h; N = 5 animals) hCG administration or 36 h after hCG administration (N = 5 animals) and stored at -80 C until analysis. Cortisol and cortisone concentrations were determined using a LC–MS system (Shimadzu Nexera-LCMS-8050) in the ONPRC Endocrine Technologies Core as previously described^38^. All samples were simultaneously analyzed in a single run for each analyte. Accuracy and intra-assay CV for cortisol was 98.0% and 5.8%, respectively.

### Immunohistochemistry (IHC) of ovarian follicles

Ovaries were collected from female rhesus macaques undergoing a controlled ovulation (COv) protocol both prior to (0 h) or after (12 h, 24 h, and 36 h) injection with a bolus of hCG as previously described^39^. The COv protocol allows for the continued development of the naturally selected follicle and the initiation of ovulation at a specified time. Ovarian sections from four different animals undergoing separate COv protocols were included at each time point for IHC of HSD11B1 and HSD11B2 expression, whereas ovaries from three different animals per time point were collected, sectioned, and used for glucocorticoid receptor (nuclear receptor 3C1, or NR3C1; Gene ID: 706,198) IHC analysis. Ovaries were fixed in 4% paraformaldehyde (PFA; Alfa Aesar, Ward Hill, MA) overnight, placed in 5% sucrose for 24 h, dehydrated in a series of ethanol solutions (50%, 70%, and 100%), embedded in paraffin, and serial sectioned as reported previously^40^. Ovarian sections (5 μm) were incubated with a rabbit polyclonal antibody that recognizes NR3C1 (Abcam, Cambridge, MA, catalog #ab3579, 1:200), HSD11B1 (Thermo Fisher, Waltham, MA, catalog #PA5-79397, 1:100), or HSD11B2 (Thermo Fisher, Waltham, MA, catalog #PA5-79399, 1:100). Primary antibodies were detected using a biotinylated anti-rabbit IgG secondary antibody (Vector Laboratories, Burlingame, CA, BA1000) and a peroxidase substrate kit (ABC Elite Kit; Vector Laboratories, Burlingame, CA, PK6100). A rabbit IgG isotype matched antibody (Abcam, Cambridge, MA, catalog #ab172730) was used as a negative control and to set the white balance in Adobe Photoshop uniformly across all images. Images for IHC were taken using a Nikon Ti-U inverted microscope with 20, 40 or 100X objectives. The above antibodies were tested via Western blot by the commercial sources to confirm specificity and showed a single band of expected size.

### Immunofluorescence (IF) of oocytes and CCs for NR3C1 detection

Oocytes and CCs were collected from female rhesus macaques undergoing COS protocols as described above at 0 h and 36 h post-hCG administration. Oocytes were also obtained from a 0 h COS and underwent in vitro maturation (IVM) for 24 h as described below to compare NR3C1 localization in oocytes post-IVM versus those that matured in vivo from a 36 h COS. Removal of the zona pellucida was accomplished by incubating oocytes in EmbryoMax Acidic Tyrode’s Solution (EMD Millipore, Burlington MA) for ~ 30 s. The oocytes were washed in 0.1% BSA (Sigma-Aldrich, St. Louis, MO) plus 0.1% Tween 20 (Sigma-Aldrich, St. Louis, MO; PBS-T) and fixed by incubation in cold 4% PFA in PBS for 20 min at room temperature (RT). Oocytes were washed with PBS-T to remove any fixative and permeabilized in 1% Triton-X (Calbiochem; Burlington, MA) for 30 min at RT. Non-specific binding sites were blocked by incubation in 4% donkey serum (Jackson ImmunoResearch Laboratories, Inc.; West Grove, PA) for 30 min at RT. Oocytes were incubated with the primary antibody for NR3C1 (Abcam, Cambridge, MA, catalog #ab3579, 1:200 in PBS-T) overnight at 4 °C and washed with PBS-T. As a negative control, oocytes were incubated with the rabbit IgG isotype control antibody described above. Primary NR3C1 antibody binding was detected by incubating samples in a donkey anti-rabbit antibody conjugated with Alexa Fluor 488 for NR3C1 (Thermo Fisher, Waltham, MA, A-21206, 1:100) for 2 h at RT. All antibodies were diluted in PBS-T + 1% donkey serum. DNA was stained with 1 μg/ml DAPI (Thermo Fisher, Waltham, MA, D1306, 1:1000) for 10 min. In between each step, the oocytes were washed with PBS-T three times for 5 min each. Oocytes were transferred to glass bottom petri-dishes (Mattek; Ashland, MA) and NR3C1 immunolocalization visualized on a Leica SP5 AOBS spectral confocal system using the 10 × and 20 × objective. Z-stacks 1–5 μM apart were imaged sequentially to avoid spectral overlap between channels.

### NR3C1 morpholino antisense oligonucleotide (MAO) design and oocyte microinjection

A NR3C1 specific MAO was designed to bind the 5′UTR upstream of the translation initiation site in the rhesus macaque *NR3C1* gene (XM_015141112.1: TGGAGTCCATCAGTGAATATCAACT), thereby inhibiting its translation. A MAO recognizing a splice site mutant of the human hemoglobin beta-chain (HBB) gene (AY605051: CCTCTTACCTCAGTTACAATTTATA) was used as a standard (STD) control. Both the NR3C1 and STD MAOs were synthesized with a 3′-carboxyfluorescein tag to aid in visualization during embryo microinjection. Oocytes were collected at 6 h or 36 h following hCG injection to rhesus macaque females undergoing a COS protocol. The MAOs were reconstituted in embryo grade water (Sigma- Aldrich, W1503) and microinjected using a CellTram vario, electronic microinjector and Transferman NK 2 Micromanipulators (Eppendorf, Hauppauge, New York, USA). The MAO concentration (0.3 mM) was chosen based on previous reports that this concentration of STD MAO did not impact blastocyst formation rates in both mice^41^ and rhesus macaques^42^.

### IVM and IVF of microinjected oocytes

Injected oocytes from the 6 h post-hCG COS were allowed to undergo IVM for 24 h to allow progression to the MI/MII stage of meiosis in 100 μl drops containing preequilibrated maturation medium (IVF Bioscience, UK, 61002 BO-IVM) and cortisol at a concentration of 64 ng/μl, which was based on the data from the LC–MS analysis of periovulatory follicle FF obtained at 36 h post-hCG administration. The IVM oocytes from the 6 h COS and the injected in vivo matured oocytes from the 36 h COS underwent conventional IVF as described above to ascertain if there were differences in NR3C1 MAO-injected oocytes matured under different conditions. Maturation and fertilization rates were calculated as follows: maturation rate = (number of mature MII oocytes/total number of oocytes) *100, fertilization rate = (number of zygotes formed/number of mature MII oocytes that underwent IVF) *100, while the cleavage and blastocyst formation rates were determined as described above.

### Cumulus cell expansion assay

Individual follicles were aspirated from separate rhesus macaques undergoing COS protocols (n = 3) 6 h post-hCG to isolate immature oocytes and their surrounding CCs. Oocytes were microinjected with either STD MAO or NR3C1 MAO as described above and re-incubated with their surrounding somatic cells (primarily CCs). Reconstructed COCs were allowed to undergo IVM in preequilibrated maturation medium with cortisol as described above for 24 h after which, CC expansion was recorded by stereomicroscopic imaging. Oocyte-CC co-culture experiments were performed similar to previously published reports, whereby the role of oocyte gap junction communication, as well as paracrine and/or juxtracrine signaling in affecting CC function was investigated^43,44^. To determine if there were differences between embryo treatment groups, hyaluronic acid (HA)synthesis, an indicator of CC expansion, was examined in reconstituted COCs by IF. Using a biotinylated HA binding protein (HABP; Sigma-Aldrich, catalog #385911, 1:200) and Alexa 488 conjugated streptavidin (Thermo Fisher Scientific, S32354, 1:200), the absence or presence of HA in CC/mGCs was visualized in Z-stacks at 4–5 μM apart by confocal microscopy as described above. Visualization of CC expansion and HABP immunostaining was performed in triplicate with similar results.

### Statistical analysis

To determine whether the FF volume in a periovulatory follicle correlated with the post-IVF outcome of the oocyte, a Chi-square (χ^2^) test was performed with *p* < 0.05 being considered significant. In the metabolomics assessment, statistical analyses were performed in ArrayStudio on natural log transformed data. Each biochemical in OrigScale was rescaled to set the median equal to 1. Values for each sample were normalized by sample volume and following normalization to sample mass, log transformation, and imputation of missing values, if any, with the minimum observed value for each compound. A mixed model ANOVA with post-hoc tests (incorporating same-subject sampling as a random effect term) was performed to identify biochemicals that differed significantly between experimental groups. Data from different run batches were run-aligned by setting the median of each group to 1 to correct for batch effect. An estimate of the false discovery rate (*q*-value) was calculated to consider the multiple comparisons that normally occur in metabolomics-based studies^37^. Analysis of the cortisol:cortisone ratio in the 74 FF samples was performed by the mixed effects model and Bonferroni multiple testing adjustment. One-way ANOVA was used to test for significant differences in concentration in FF between 0 and 36 h COS cycles by LC–MS. The uncleaved, cleavage, and blastocyst formation rate was calculated as follows: uncleaved rate = (number of oocytes that remained unfertilized or those that appeared to fertilize, but did not cleave/number of mature MII oocytes that underwent IVF) *100, cleavage rate = (number of embryos that cleaved/number of zygotes formed) * 100, and blastocyst formation rate = (number of blastocysts formed/number of cleaved embryos) *100 and compared between follicles that had greater or less than 35 μl FF volume.

## Results

### Classification of post-fertilization developmental outcomes and association with FF volume

Both the oocyte and FF were collected from individual follicles of rhesus macaque females (N = 17) undergoing COS protocols to compare the FF metabolites to the corresponding oocyte’s developmental potential post-IVF. Isolated oocytes (N = 255) and the corresponding FF samples were categorized as uncleaved, cleavage stage, and blastocyst as explained in the methods. A higher blastocyst formation rate and lower cleavage rate was observed from the oocytes aspirated from follicles with a FF volume greater than 35 μl compared to a lower blastocyst formation rate and higher cleavage rate detected in follicles with a FF volume less than 35 μl. The uncleaved rate, however, remained unchanged (Supplementary Fig. S2). A chi-square test (χ^2^) revealed a significant correlation between FF volume and the post-IVF outcomes of resident oocytes (χ^2^ = 15.921, *p* < 0.001), which suggested that FF volume can be used as an indicator of oocyte competency.

**Figure 1:**
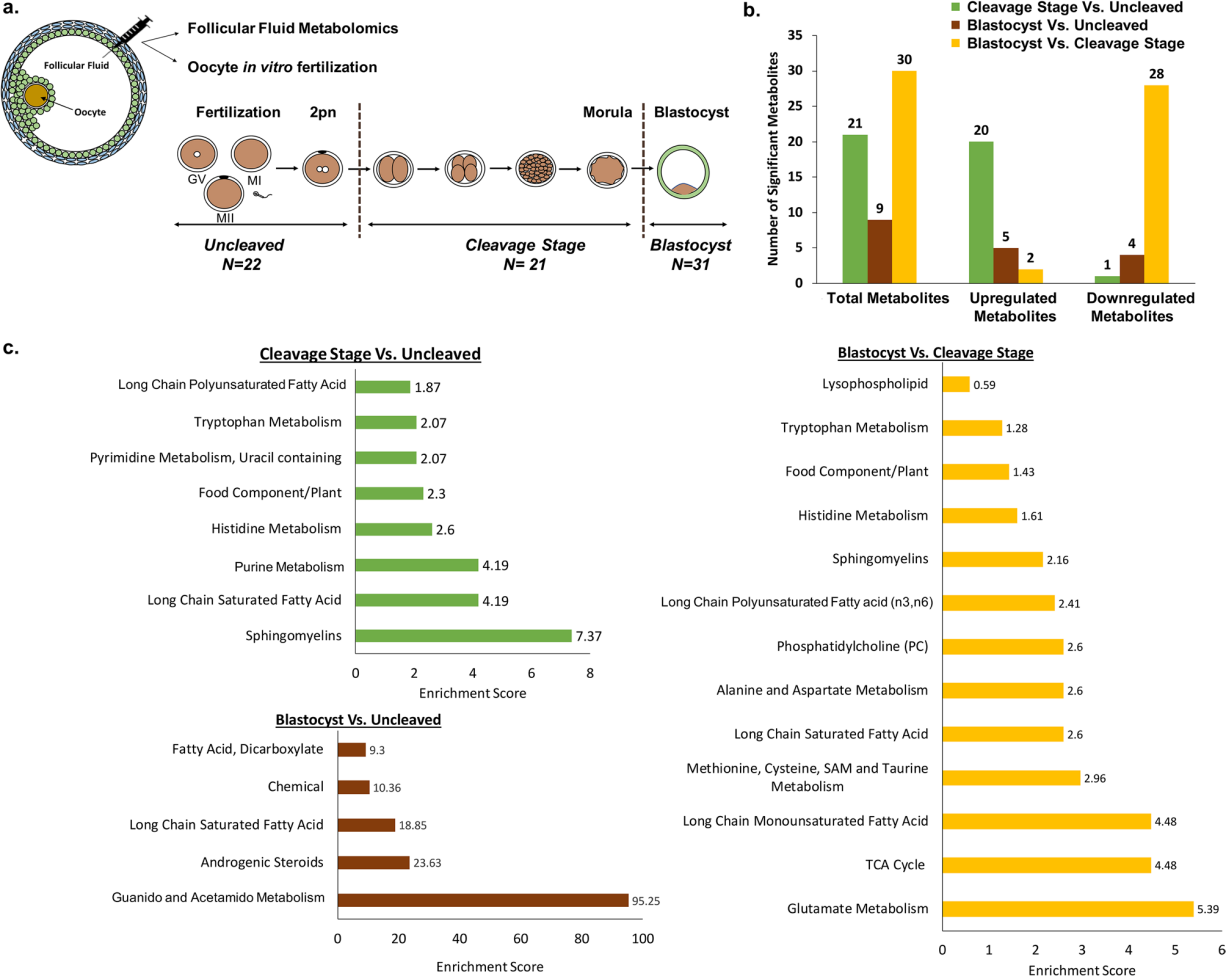
Metabolomics assessment of follicular fluid constituents. (**a**) Schematic depicting the experimental design of the study with the collection of FF for metabolomics analysis and the corresponding oocyte for IVF. Resultant embryos were classified as uncleaved (N = 22), cleavage stage (N = 21), or blastocyst (N = 31). (**b**) The total number of significant metabolites (*p* ≤ 0.05) that were upregulated or downregulated in the FF between the embryo groups was determined through pairwise comparisons. Note that the majority of metabolites that were statistically different between the blastocyst and cleavage stage embryo groups were downregulated in blastocysts. (**c**) Pathway enrichment analysis of the metabolites in the FF shown as pairwise comparisons identified multiple fatty acid and metabolism pathways as well as the TCA cycle components that were different between the embryo groups. An enrichment score of greater than 1 indicates that the pathway is enriched in the first group listed compared to the second group and a value of less than 1 indicates that the pathway is enriched in the second group listed versus the first group. Enrichment score = (k/m)/((n-k)/(N-m)) where m = number of metabolites in the pathway, k = number of significant metabolites in the pathway, n = total number of significant metabolites and N = total number of metabolites.

Out of the 255 oocytes that were obtained from individual follicle aspirations, fertilized via conventional IVF, and underwent pre-implantation development, 74 FF samples with a volume of 40 μl or greater were chosen for further analysis and categorized as uncleaved (N = 22), cleavage stage (N = 21), and blastocysts (N = 31) as defined in the methods and Fig. 1a. To minimize the bias towards any single female, we selected embryos from as many rhesus macaque females as possible, including 9 females in the uncleaved group, 12 females in the cleavage stage group, and most importantly, 14 females in the blastocyst group. The distribution of animals in each group is represented in Supplementary Fig. S3.

### Metabolomics analysis of FF and identification of signaling pathways indicative of oocyte competency

Non-targeted metabolomics profiling was performed on the corresponding 74 FF samples to determine whether there were differences in the intrafollicular microenvironment between groups. While two separate metabolomics assessments were initially performed for each year, the final results were batch-normalized to generate a dataset with greater statistical power and avoid potential heterogeneity amongst females. A total of 382 biochemicals were identified in the FF, of which 348 had a known identity (named biochemicals) and 34 had both unknown structural identity and biological function (unnamed biochemicals). Statistically significant differences of FF biochemicals were noted between the three embryo groups, with the greatest difference observed in FF obtained from follicles that yielded an oocyte that arrested at the cleavage stage versus those that progressed to the blastocyst stage (Fig. 1b). Interestingly, the majority (~ 93%; N = 28/30) of these metabolites were downregulated in the blastocyst group compared to those embryos that arrested at the cleavage stage.

After combining all the identified biochemicals from each embryo category, we performed pathway enrichment analysis, which revealed entire molecular pathways that were differentially represented between embryo groups (Table 1). As shown in Fig. 1c, the sphingomyelin pathway was the most enriched in the FF from follicles with oocytes that did not cleave following IVF versus FF associated with oocytes that produced arrested cleavage stage embryos. With the exception of uracil-derived orotate (Supplementary Fig. S4), the majority of these metabolites were at higher levels in follicles yielding oocytes that led to cleaved embryos. In contrast, the guanido and acetamido amino acid metabolism pathways were enriched in the FF containing oocytes that did not cleave relative to those that developed into blastocysts. The greatest pathway enrichment differences observed by pairwise comparison were between embryos that arrested at the cleavage stage versus those that formed blastocysts, which included long chain monosaturated fatty acid, the tricarboxylic acid cycle (TCA), and glutamate metabolism (Fig. 1c).

**Table 1:** List of metabolite-based pathways that significantly differ between embryo groups.

Number	Super pathway	Sub pathway	Cleavage-stage : uncleaved ratio
1	Amino acid	Histidine metabolism	**1.85**
2		Tryptophan Metabolism	**1.33**
3	Peptide	Dipeptide	**1.35**
4	Lipid	Long Chain Saturated Fatty Acid	**1.11**
5		Long Chain Polyunsaturated Fatty Acid (n3 and n6)	**1.43**
6		Fatty Acid Metabolism (also BCAA Metabolism)	**1.39**
7		Phosphatidylethanolamine (PE)	**1.41**
8		Phosphatidylethanolamine (PE)	**1.37**
9		Phosphatidylinositol (PI)	**1.26**
10		Phosphatidylinositol (PI)	**1.24**
11		Sphingomyelins	**1.31**
12		Sphingomyelins	**1.28**
13		Androgenic Steroids	**1.73**
14	Nucleotide	Purine Metabolism, (Hypo)Xanthine/Inosine	**1.63**
15		Pyrimidine Metabolism, Orotate containing	*0.71*
16		Pyrimidine Metabolism, Uracil containing	**2.24**
17		Purine and Pyrimidine Metabolism	**2.08**
18	Co-factors and vitamins	Ascorbate and Aldarate Metabolism	**1.27**
19	Xenobiotics	Food Component/Plant	**2.47**
20	Unnamed compounds	Unnamed compounds	**1.4**
21		Unnamed compounds	**1.81**

Number	Super pathway	Sub pathway	Blastocyst : uncleaved ratio
1	Dipeptide	Guanidino and Acetamido Metabolism	*0.78*
2	Lipid	Long Chain Saturated Fatty Acid	*0.83*
3		Fatty Acid, Dicarboxylate	*0.63*
4		Androgenic Steroids	**1.76**
5	Xenobiotics	Chemical	**1.36**
6	Unnamed compounds	Unnamed compounds	**1.36**
7		Unnamed compounds	**1.93**
8		Unnamed compounds	**1.35**
9		Unnamed compounds	*0.66*

Number	Super pathway	Sub pathway	Blastocyst : cleavage-stage ratio
1	Amino acid	Alanine and Aspartate Metabolism	*0.69*
2		Glutamate Metabolism	*0.71*
3		Glutamate Metabolism	*0.55*
4		Histidine metabolism	*0.57*
5		Tryptophan metabolism	*0.76*
6		Methionine, Cysteine, SAM and Taurine	*0.56*
7		Methionine, Cysteine, SAM and Taurine	*0.77*
8		Creatine Metabolism	*0.87*
9		Polyamine Metabolism	**1.22**
10	Peptide	Dipeptide	*0.78*
11	Energy	TCA cycle	*0.66*
12		TCA cycle	*0.63*
13	Lipid	Medium Chain Fatty Acid	*0.68*
14		Long Chain Saturated Fatty Acid	*0.88*
15		Long Chain Monounsaturated Fatty Acid	*0.85*
16		Long Chain Monounsaturated Fatty Acid	*0.7*
17		Long Chain Polyunsaturated Fatty Acid (n3 and n6)	*0.73*
18		Long Chain Polyunsaturated Fatty Acid (n3 and n6)	*0.66*
19		Fatty Acid Metabolism (also BCAA Metabolism)	*0.76*
20		Fatty Acid Metabolism (Acyl Carnitine, Medium Chain)	**1.5**
21		Eicosanoid	*0.47*
22		Phospholipid Metabolism	*0.84*
23		Phosphatidylcholine (PC)	*0.65*
24		Phosphatidylethanolamine (PE)	*0.77*
25		Lysophospholipid	*0.73*
26		Spingomyelins	*0.8*
27	Nucleotide	Purine Metabolism, Adenine containing	*0.65*
28		Purine and Pyrimidine Metabolism	*0.44*
29	Co-factors and Vitamins	Ascorbate and Aldarate Metabolism	*0.82*
30	Xenobiotics	Food Component/Plant	*0.61*

Bold indicates significant difference (*p* ≤ 0.05) between the groups shown; metabolite ratio of ≥ 1.00. Italics indicates significant difference (*p* ≤ 0.05) between the groups shown, metabolite ratio of < 1.00.

**Figure 2:**
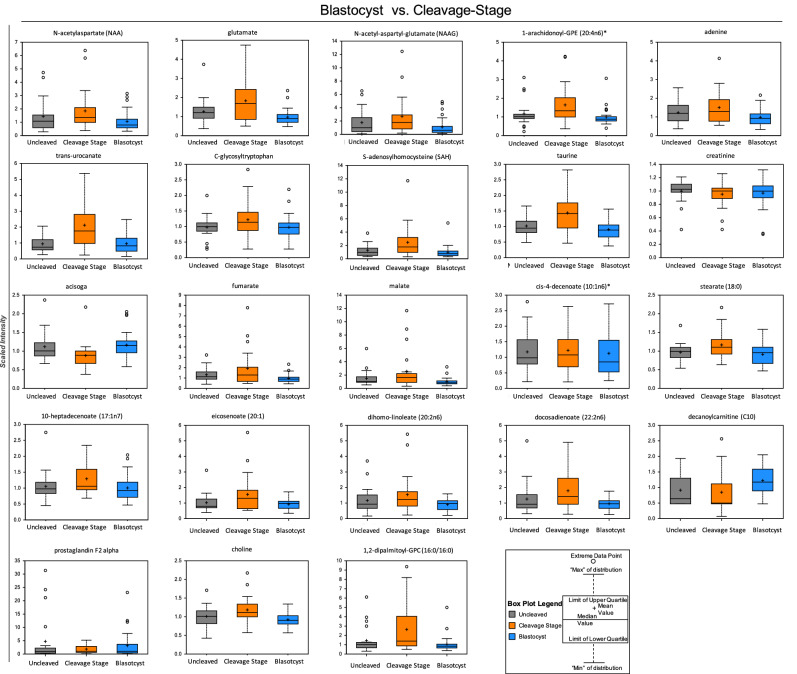
Representative box plots of metabolites that were significantly different between FF samples belonging to the blastocyst versus cleavage stage embryo group (*p* < 0.05). The y-axis reflects the scaled intensity of each metabolite in uncleaved (gray) cleavage stage (orange) and blastocysts (blue). The original data generated by the area under curve of the MS peaks was used for median-scaling, determining the minimum and maximum of data distribution, and identification of outliers. Note that with the exception of acisoga, an end product of polyamine metabolism, and the fatty acid lipid molecule, decanoylcarnitine (C10), most of the metabolites were downregulated in blastocysts compared to cleavage stage embryos.

Box plots of the significantly different metabolites between the blastocyst and cleavage stage arrested groups were constructed and are shown in Fig. 2, whereas the uncleaved versus cleaved or blastocyst comparisons are shown in Supplementary Fig. S4. In addition, there were several metabolites belonging to same molecular pathway that showed a trend between the embryo groups, but were not statistically significant (Supplementary Fig. S5). These metabolites included alanine, threonine, asparagine, tyrosine, proline, and methionine from the amino acid pathway and prostaglandin E2, 1-linoleoyl-GPC, 1-oelyl-GPC, 1-palmitoyl-GPC, 3-hydroxyadipate of the lipid pathway, which should be further investigated in additional cohorts of individual follicles.

### Correlation between glucocorticoid levels in FF and embryo developmental potential

**Figure 3:**
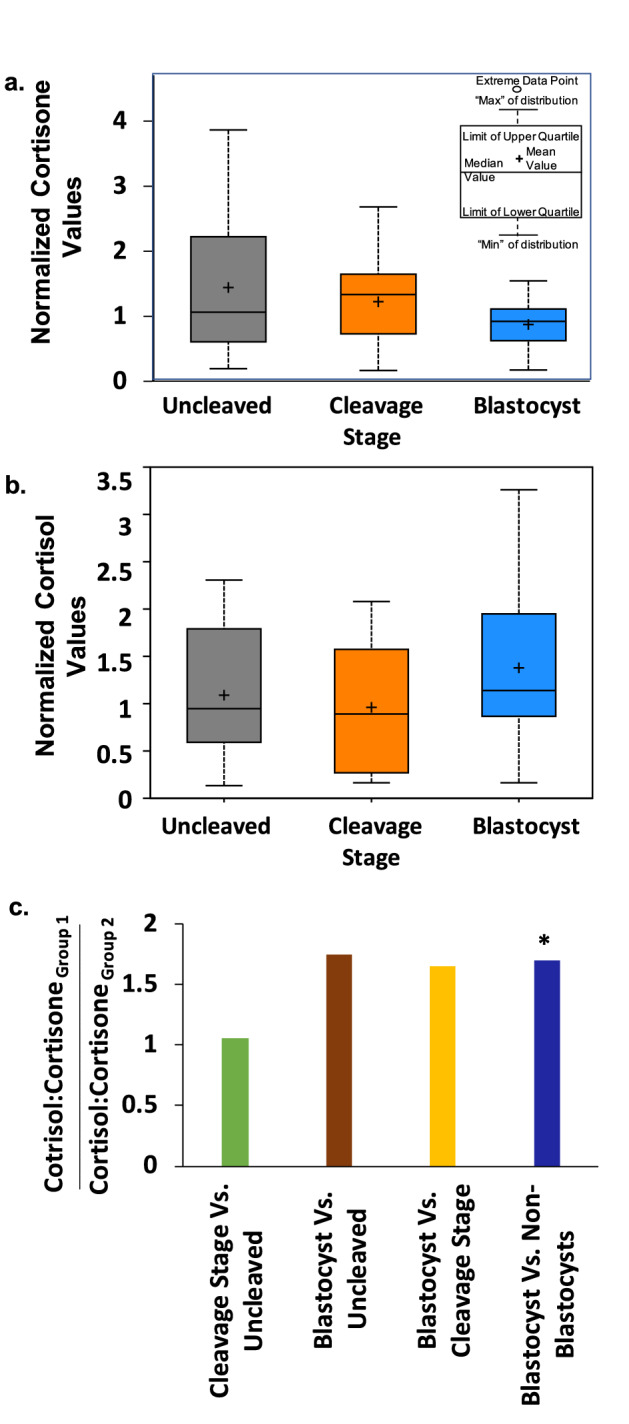
Glucocorticoid levels in FF are indicative of oocyte competency. Box plots depicting the normalized values of (**a**) cortisone and (**b**) cortisol in the FF of each corresponding embryo group. (**c**) The ratio of cortisol to cortisone in the FF shown by a pairwise comparison between the embryo groups. Note that the cortisol to cortisone ratio is significantly higher in the FF of the blastocyst versus non-blastocyst groups (*; *p* = 0.0283).

**Figure 4:**
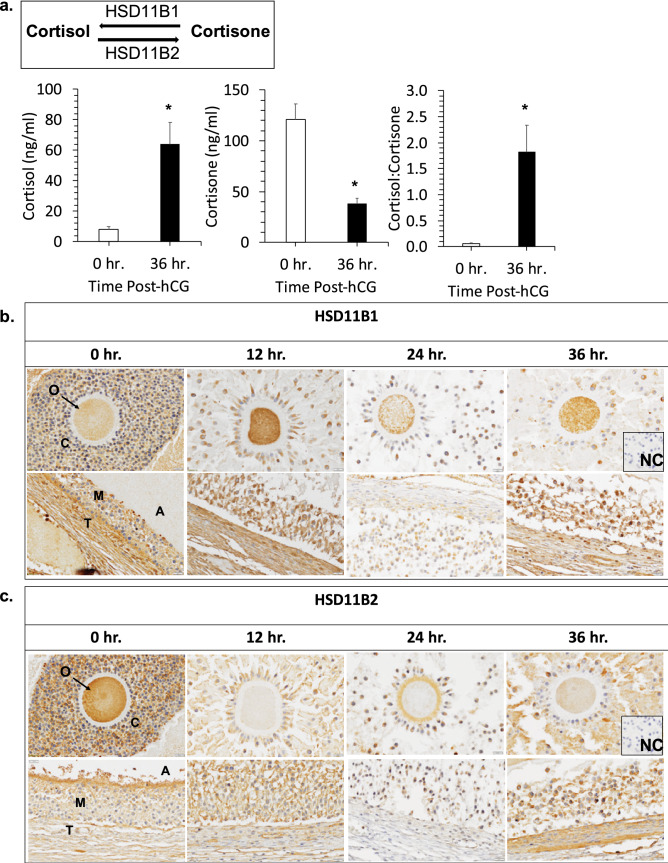
Glucocorticoids and the enzymes that metabolize glucocorticoids are present in the rhesus macaque periovulatory follicle. (**a**) LC–MS/MS analysis of cortisol and cortisone concentrations in the FF obtained from rhesus macaque follicles between pre- (0 h) and 36 h post-hCG administration. A statistically significant increase in cortisol (*p = 0.0137) and decrease in cortisone (*p = 0.0110), with a corresponding increase in the cortisol to cortisone ratio (*p = 0.0256), was observed following hCG injection. (**b**) IHC of HSD11B1 and (**c**) HSD11B2 immunolocalization in the rhesus macaque periovulatory follicle pre- (0 h) as well as 12 h, 24 h and 36 h post-hCG administration. The images shown are representative of N = 4 ovaries obtained from separate animals undergoing a COv protocol at each of the times indicated. Note the overall increase in HSD11B1, as well as a concomitant decrease in HSD11B2 expression, in the ovarian follicle prior to hCG administration and with increasing time after the ovulatory stimulus. O = oocyte, C = cumulus cells, A = antrum, T = theca cells, M = mural granulosa cells and NC = negative control.

Among the metabolites that significantly differed between the three embryo groups, the levels of the glucocorticoid metabolites, cortisol and cortisone, in FF were strongly associated with the developmental potential of the resident oocyte in both the individual and merged metabolomics datasets. Other than the glucocorticoids, there were no additional steroids whose levels differed significantly between the embryo groups. Cortisone is biologically inactive because it has low affinity for NR3C1, whereas cortisol binds NR3C1 at high affinity to activate downstream signaling events. While higher FF cortisol levels were detected within follicles associated with an oocyte that formed a blastocyst (Fig. 3a), higher cortisone levels were detected within follicles with oocytes that remained uncleaved or arrested in development post-fertilization (Fig. 3b). A pairwise comparison of the FF ratio of cortisol: cortisone and showed that the ratio of these glucocorticoids in the FF associated with an oocyte that formed a blastocyst was significantly higher (*p* = 0.0283) than the oocyte that did not form a blastocyst (Fig. 3c) Based on these findings, we reasoned that the cortisol:cortisone ratio in FF is an important determinant of oocyte competency and that the intrafollicular levels of cortisol and cortisone during the periovulatory interval warrant further investigation. Thus, we obtained FF from rhesus macaque females undergoing a COS that did not receive hCG (0 h) or FF collected 36 h after hCG administration, which was used for LC–MS assessment of cortisol and cortisone levels. As shown in Fig. 4a, cortisone levels significantly declined, whereas cortisol levels significantly increased after hCG administration, supporting a role for glucocorticoid signaling in the periovulatory process.

### Enzymatic interconversion of glucocorticoids in the periovulatory follicle

The active and inactive forms of glucocorticoids are interconvertible through the action of two enzymes, HSD11B1 and HSD11B2^45^. HSD11B1 converts cortisone to cortisol, which binds to and activates NR3C1, whereas HSD11B2 converts cortisol to cortisone, a compound that has low to minimal affinity for NR3C1^46,47^. We previously demonstrated by microarray analysis in female rhesus macaques undergoing a COv protocol that HSD11B1 and HSD11B2 mRNA levels in the periovulatory follicle significantly increase and decrease, respectively, after hCG administration^48^. In order to determine the cellular localization of the two enzymes in the follicle during the periovulatory interval, IHC was performed on ovaries removed from animals undergoing a COv protocol prior to (0 h) or 12 h, 24 h, and 36 h after hCG administration (N = 4 at each time point). As shown in Fig. 4b, HSD11B1 was expressed in the oocyte, CCs, and theca cells of ovarian follicles both pre- and post-hCG administration. There was also an apparent increase in perinuclear mGC expression of HSD1B1 over time, with the highest level of expression detected at 36 h post-hCG administration. In contrast, positive HSD11B2 immunostaining (Fig. 4c) was observed in the oocyte and the periphery of the CCs before hCG administration (0 h), but diminished after hCG administration. While mGCs expressed HSD11B2 at 36 h post-hCG administration, HSD11B2 immunolabeling was lower in theca cells relative to the staining intensity of HSD11B1. Thus, an increase in HSD11B1, with a corresponding decrease in HSD11B2 staining intensity, in the follicle correlated with the increased cortisol:cortisone ratio observed in FF following an ovulatory stimulus.

### Expression and localization of NR3C1 in oocytes and ovarian follicles

**Figure 5:**
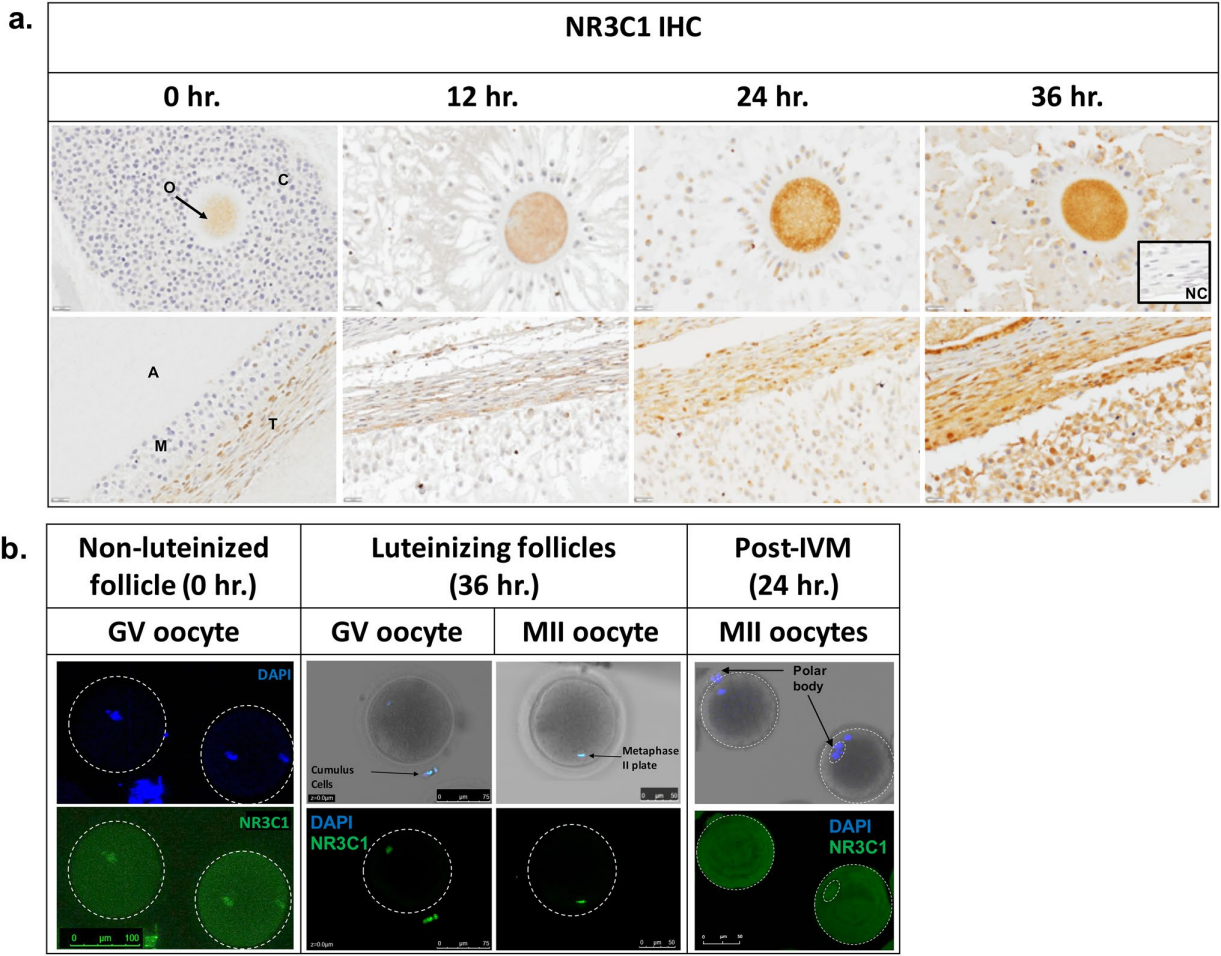
NR3C1 localizes to oocytes and somatic cells in the rhesus macaque periovulatory follicle**.** (**a**) NR3C1 IHC was performed on rhesus macaque periovulatory follicles pre- (0 h) and 12 h, 24 h and 36 h post-hCG administration. Positive immunolabeling (brown) was detected in the oocyte, CCs, mGCs, as well as the theca cells lining the follicle wall. There was an apparent reduction in oocyte-specific NR3C1 staining at 12 h post-hCG and the highest intensity of NR3C1 staining was detected in all cell types at 36 h post-hCG administration. The images shown are representative of N = 3 ovaries obtained from individual animals undergoing a COv protocol at each of the times indicated. O = oocyte, C = cumulus cells, A = antrum, T = theca cells, M = mural granulosa cells, and NC = negative control. (**b**) NR3C1 IF in rhesus macaque oocytes obtained from follicles before (0 h) and 36 h after hCG administration demonstrated NR3C1 localization (green) in both the cytoplasm and nucleus of non-luteinized GV oocytes, but only in the nucleus of luteinizing GV oocytes and CCs that remained attached. Note that NR3C1 also localized to the metaphase plate of mature MII oocytes and that GV oocytes from non-luteinized follicles that reinitiated meiosis by IVM exhibited only cytoplasmic NR3C1 expression. DAPI (blue) was used as a marker for DNA.

Our next objective was to determine which cell types are potentially responsive to glucocorticoid action by immunolocalizing NR3C1 in the rhesus macaque periovulatory follicle. Ovarian tissues obtained from female rhesus macaques undergoing a COv protocol at the same time points pre- and post-hCG injection as described above were examined by IHC (N = 3 at each time point). NR3C1 immunostaining was observed in the oocyte, CCs, mGCs, as well as the theca cells lining the follicle wall (Fig. 5a). There was a reduction in oocyte-specific NR3C1 staining at 12 h post-hCG and the highest intensity of NR3C1 staining was detected in all cell types at 36 h post-hCG administration. Since the presence of NR3C1 was evident in the oocyte, we further assessed its expression within immature and mature oocytes by IF. GV oocytes were collected from non-luteinized, pre-ovulatory follicles (0 h COS, N = 18) and NR3C1 immunolocalization was observed in both the cytoplasm and nucleus of immature oocytes (Fig. 5b). However, 36 h after hCG administration, NR3C1 expression was only observed in the nucleus of GV oocytes and CCs that remained attached (N = 23). Unexpectedly, NR3C1 also localized to the metaphase plate of mature MII oocytes. The GV oocytes from non-luteinized follicles that reinitiated meiosis by IVM did not exhibit nuclear localization of NR3C1 (N = 16).

### Functional assessment of NR3C1 action

After determining the expression patterns of NR3C1, HSD11B1, and HSD11B2 in the primate periovulatory follicle, we next sought to ascertain the functional role of NR3C1 and the glucocorticoid signaling pathway in rhesus macaque oocyte function, maturation, fertilization, and pre-implantation embryo development. Thus, a MAO designed to target the 5′UTR of NR3C1 was microinjected into oocytes using a non-targeting MAO as a negative control. Initially, GV, MI, and MII oocytes aspirated 36 h after hCG administration were injected with either STD MAO (N = 8) or the NR3C1 MAO (N = 15), and NR3C1 knockdown was assessed by IF. Within 24 h of microinjection, up to 80% of the NR3C1 MAO injected oocytes did not contain any discernable NR3C1 expression (Supplementary Fig. S6a). NR3C1 staining was not reduced in oocytes receiving the STD MAO, demonstrating that the knockdown of NR3C1 in oocytes using a MAO approach was robust and specific.

Once we confirmed efficient NR3C1 knockdown in oocytes, NR3C1 involvement in fertilization and the initial cleavage divisions leading up to blastocyst formation was also assessed in oocytes collected from a 36 h post-hCG COS cycle. Comparison of oocytes injected with NR3C1 MAO (N = 43; 38 MI/MIIs and 5 GVs) or STD MAO (N = 38; 33 MI/MIIs and 5 GVs) showed no significant differences in maturation, fertilization, cleavage or blastocyst formation rates (Supplementary Fig. S6b). To determine if blocking NR3C1 expression closer to the time that re-initiation of meiosis occurs in vivo would have an impact on oocyte maturation, fertilization, or embryonic development, oocytes were collected 6 h after hCG administration during a COS protocol. As expected, mostly GV oocytes were obtained at this time point, but a small number of maturing MI oocytes were also collected. These oocytes were injected with STD MAO (N = 30; 27 GVs and 2 MIs) or NR3C1 MAO (N = 28; 27 GVs and 1MI) and cultured in the absence or presence of cortisol. However, no significant differences in the completion of meiosis, fertilization, or cleavage rates were detected between treatment groups, which suggested that NR3C1 was not directly involved in the resumption of oocyte meiosis in vitro or subsequent development (Supplementary Fig. S6c).

### NR3C1 knockdown in oocytes has indirect effects on CC expansion

**Figure 6:**
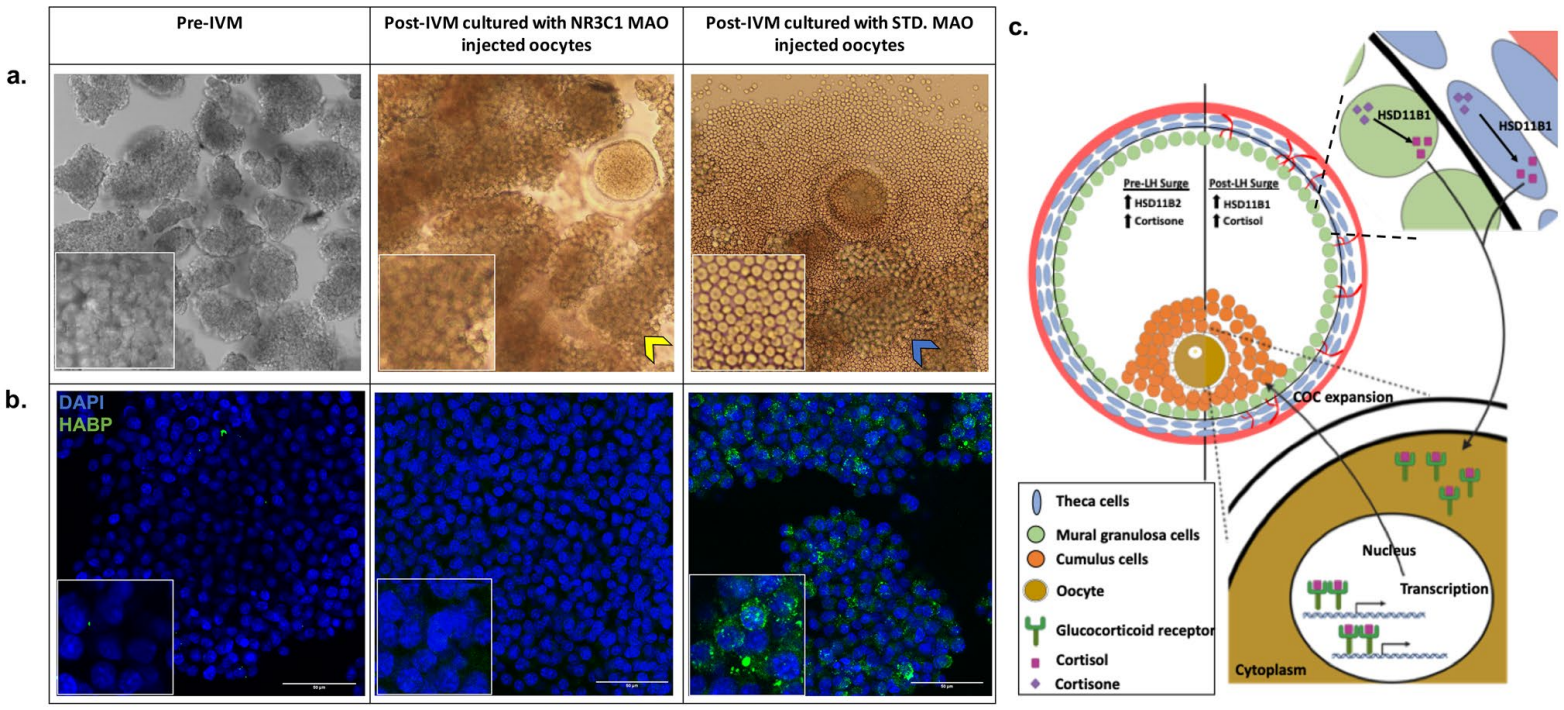
NR3C1 is essential for cross-communication between the oocyte and the surrounding CCs. (**a**) Stereomicroscope image of CC morphology immediately after aspiration and prior to IVM (left) and in the presence of a NR3C1 MAO-injected oocyte (middle) versus an STD MAO-injected oocyte (right) after IVM. Note the clumped CCs (image insets) in the pre-IVM and NR3C1 MAO co-cultures (yellow arrowhead) compared to the single CCs visible following expansion (blue arrowhead) (**b**) IF detection of HA using HABP confirmed a lack of expansion in pre-IVM CCs and those incubated with NR3C1 MAO-injected oocytes as evidenced by the little to no HABP (green) immunostaining in CCs also stained with DAPI (blue). In contrast, robust HABP immunostaining was observed in the CCs co-cultured with STD MAO-injected oocytes. (**c**) A proposed model for NR3C1-dependent bidirectional communication between the oocyte and somatic cells. The increased relative level of HSD11B1 to HSD11B2 in the mGCs/CCs (green) and the theca cells (blue) convert cortisone to cortisol post-LH surge, with cortisol binding to NR3C1 in the oocyte. Nuclear translocation of cortisol bound NR3C1, activation of transcription, and further unknown downstream events leads to COC expansion that takes place during meiotic maturation of the oocyte.

Although there was no effect of NR3C1 knockdown on oocyte function, it was noted that the CCs obtained as part of the COCs exhibited a different morphology based on whether they were incubated with oocytes microinjected with the NR3C1 MAO versus those injected with STD MAO. At 6 h post-hCG administration, CCs have not yet undergone expansion and remain closely associated with one another relative to the expanded CCs retrieved 36 h after hCG administration. In the presence of the NR3C1 MAO injected oocytes, the CCs did not expand even after 30 h in culture and remained tightly associated with one another similar to the CCs obtained immediately after follicular aspiration (Fig. 6a). In contrast, the CCs cultured with the STD MAO injected oocytes expanded so that individual cells, rather than cell clumps, were observed. To confirm that NR3C1 knockdown in the oocyte was having an effect on the processes important for CC expansion, we performed a similar NR3C1 MAO or STD MAO microinjection experiment whereby each injected oocyte was cultured with its corresponding CCs in IVM media for 24 h. CC cultures were stained with HABP, a protein that binds to secreted HA, which serves as a definitive marker for CC expansion^49^. While HA localized to the extracellular space in between the CCs of STD MAO injected oocytes, the CCs cultured with the NR3C1 MAO injected oocytes were negative for HA staining and appeared similar to the pre-IVM CCs/mGCs (Fig. 6b). This suggests that NR3C1 knockdown in oocytes has indirect effects on COC function by preventing the expansion of CCs via paracrine signaling during oocyte maturation and the resumption of meiosis.

## Discussion

Circulating and intrafollicular derived factors are critical for ovulatory follicle development in humans, non-human primates (NHPs), and rodents^5,7–14^ and thus, the FF and its constituents are likely a reflection of the resident oocyte’s capacity for fertilization and development. Indeed, we observed in this study that the volume of FF is a significant indicator of whether a fertilized oocyte will cleave and successfully form a blastocyst, which supports previous reports of a positive correlation between FF volume and IVF outcome^50,51^. Because a minimum volume of 40 µl of FF was required for metabolomics analysis, it should be noted that there is likely an inherent and unavoidable selection bias in our study for follicles that have a higher probability of yielding an oocyte capable of fertilization and forming a blastocyst. However, despite this bias, a number of metabolites whose FF levels exhibited a significant association with oocyte fertilization and blastocyst development were still identified. Previous genomics, proteomics and metabolomics studies reported mixed results in correlating follicular contents with pre-implantation embryo development^9,29,52–60^. In the metabolomics studies performed to date, techniques capable of discerning a limited number of known metabolites were primarily utilized and/or a pool of FF from a heterogenous population of multiple follicles was analyzed. By pooling the FF, it is not possible to directly correlate identified factors with the oocyte’s potential to undergo fertilization, cleavage, and blastocyst formation. Here, we performed an unbiased metabolomics analysis of FF samples from individual rhesus macaque periovulatory follicles to evaluate metabolites that represent the sum of all genomic, transcriptomic, and proteomic processes occurring in the follicle. Our approach also gave us the ability to directly correlate the metabolomics profile of each ovarian follicle to the developmental outcome of the corresponding resident oocyte post-IVF through the blastocyst stage. To the best of our knowledge, this study is the first to perform a comprehensive assessment of the metabolome in the FF of a large number of individual developing follicles using a unique and translationally relevant NHP model*.*

Over the past several years, nuclear magnetic resonance (NMR) and tandem mass spectrometry (MS/MS) were used to characterize the FF metabolome. MS/MS, which was used in this study, is more sensitive than NMR based approaches^61^ and the addition of different chromatographic methods has increased the number of metabolites that can be detected in a sample. Nonetheless, NMR identified glucose, choline, and creatine in FF as possible biomarkers of mature oocytes, while glucose, citrate and valine appeared important for early embryogenesis^29^. However, there was no significant association between these molecules and any particular stage of pre-implantation development. A meta-analysis of progesterone in FF indicated that it might be a useful biomarker for successful fertilization, but it also failed to explain the competency of a fertilized oocyte to form a blastocyst^62^. Unlike these factors, lower amino acid turnover in the spent media of 4- to 8-cell embryos was shown to be significantly correlated with development to the blastocyst stage, whereas a higher amino acid turnover rate was more likely to result in embryo arrest^23,24^. It was proposed that the high turnover of amino acids is an indicator of the metabolic stress involved in repairing the damage to DNA, RNA or proteins during development^63^. From our analysis, we were able to confirm this finding by showing that the majority of the FF samples with higher amino acid quantities belonged to the arrested embryos, rather than the uncleaved or blastocyst groups. Moreover, the assessment of individual amino acids revealed an abundance of glutamate metabolism in the FF of oocytes that formed blastocysts. Glutamate is a main component of the fluid in the human fallopian tube^64^ and our results concur with other reports suggesting that supplemental glutamate in bovine embryo culture media promotes blastocyst formation^65^. Besides amino acid differences, we also observed an enrichment of metabolites associated with the TCA cycle in the blastocyst group, which supports previous findings that glucose metabolism and pyruvate uptake may serve as markers for embryo viability^23,66,67^. Overall, the majority of statistically significant metabolites were downregulated in the FF of embryos that formed a blastocyst, providing support for the “quiet embryo hypothesis” that states the importance of endogenous resources over nutrient supplementation in embryo survival^68^.

From our metabolomics analysis of FF, numerous compounds were identified that showed significantly different levels between embryo groups and should be further investigated in future studies to determine how each metabolite and their associated pathways affect oocyte fertilization and post-fertilization embryogenesis. Based on the finding that the level of glucocorticoids in FF was strongly associated with the developmental potential of the resident oocyte in both the individual and merged metabolomics assessment and because there is relatively little information regarding the role that corticosteroid signaling plays in the periovulatory follicle, we chose to focus on cortisol and cortisone in this study. While it was previously reported that cortisol and cortisone, as well as the HSD11B1 and HSD11B2 enzymes responsible for their interconversion, are present in bovine and human ovaries^69–73^, there are no studies indicating an association between FF glucocorticoid levels and oocyte competency. The presence of significant levels of cortisol in FF obtained from rhesus macaque periovulatory follicles was previously reported and both HSD11B1 and HSD11B2 mRNA are known to be expressed in mGCs^73^. In response to hCG administration, it was also shown that HSD11B1 mRNA expression increased, while HSD11B2 mRNA expression decreased, in rhesus macaque periovulatory follicles as early as 12 h post-hCG injection and was retained up to 36 hr^48^. However, neither of these studies examined which follicular cell type(s) might be responsible for these changes in enzyme expression and whether it correlated with the levels of cortisol and cortisone in the FF. Here, we show that the HSD11B1 and/or HSD11B2 localize to all sub-compartments of follicle, including the oocyte, GCs, CCs, and theca cells, and that HSD11B1 expression increased with a corresponding decrease in HSD11B2 expression post-hCG administration. Thus, the changes in the FF cortisol to cortisone ratio in the follicle appears to be directly linked to the relative expression levels of both enzymes.

With the exception of NHPs, the expression of the NR3C1 has been reported in the ovary of several species^70,74–76^. Most recently, NR3C1 was shown to be expressed in both murine and porcine oocytes and CCs, but there were species-specific differences noted in oocyte sensitivity to glucocorticoids that was mediated by NR3C1 in pigs and NR3C1-independent in mice^77,78^. There are two NR3C1 splice variants that arise from the differential use of exon 9, yielding an alpha and beta isoform^79^. Since the majority of the nucleotide and amino acid sequence overlaps between the two, we cannot distinguish the relative level of expression from each of the isoforms. Our study is the first to show the presence and localization of NR3C1 within the oocyte and surrounding somatic cells of naturally selected primate follicles. We also demonstrate that NR3C1 expression is retained in oocytes in vitro and appears to change its intracellular location from the cytoplasm to the nucleus during maturation. NR3C1 is a member of the nuclear receptor superfamily of ligand-dependent transcription factors and translocates from the cytoplasm to the nucleus after ligand binding. Once in the nucleus, NR3C1 binds to glucocorticoid response elements (GREs) in DNA to control transcription and activate downstream signaling^80^. We observed a similar cytoplasmic to nuclear translocation of NR3C1 in rhesus macaque oocytes 36 h after hCG administration and, unexpectedly, NR3C1 also localized to the metaphase plate of MII oocytes that matured in vivo. In contrast, IVM MII oocytes retained NR3C1 expression only in the cytoplasm. It is well-established that the oocytes matured via IVM have increased likelihood of aneuploidy^81,82^ and lower IVF success rates than those that are matured *in vivo*^83–85^, accounting for its limited use (1–2%) in human IVF cycles (cdc.gov/art). Our results suggest that a lack of NR3C1 nuclear translocation and activation in oocytes matured in vitro might also disrupt downstream signaling events that contribute to lower IVF success.

To complement our expression studies and further interrogate NR3C1 function in primate oocytes, we performed NR3C1 knockdown to determine if glucocorticoid signaling is necessary for the establishment or maintenance of oocyte competency. Reduced NR3C1 expression had no impact on oocyte maturation, fertilization, or subsequent development. It was observed, however, that the expansion of CCs did not occur when they were co-incubated with NR3C1 MAO-injected oocytes compared to the CC expansion observed with control MAO-injected oocyte co-incubation. During cumulus-oocyte expansion, HA is released by the CCs into the extracellular space surrounding the oocyte to form a 3-dimensional network of macromolecules that facilitates gradual CC dispersion and extracellular matrix reorganization^7,86^. Using HABP, we observed HA localization to the extracellular space between CCs that had been co-cultured with STD MAO injected oocytes, but not those mGCs and CCs cultured in the presence of the NR3C1 MAO injected oocytes. Our findings suggest that HA production by the CCs was inhibited by oocyte NR3C1 deficiency and that NR3C1 mediated signaling in the oocyte indirectly regulates cumulus expansion. Thus, by binding to and activating NR3C1, glucocorticoids within the periovulatory follicle are likely involved in regulating the cross communication between the oocyte and somatic cells that is essential for cumulus-oocyte function (Fig. 6c).

Given conflicting findings on whether cortisol improves IVF treatment over the past three decades^87–89^ and that cortisol concentrations significantly vary in women after a 36 h stimulation, additional studies are needed to determine the precise relationship between FF cortisol and oocyte competency. Based on the results presented here, we would suggest that focus be placed on assessing the entire follicular microenvironment, consisting of the CCs, mGCs and the FF, to unveil potential paracrine signaling and oocyte-somatic cell crosstalk that is important for ovarian function. Apart from cortisol, there were other metabolites identified in FF whose concentration indicated an association with oocyte competency, but their biological function is currently unknown and additional studies are required to determine the importance of these molecules.

## Supplementary Information

Supplementary Information 1.

Supplementary Information 2.

Supplementary Information 3.

Supplementary Information 4.

Supplementary Information 5.

Supplementary Information 6.
