# Author Correction: Metabolomics analysis of follicular fluid coupled with oocyte aspiration reveals importance of glucocorticoids in primate periovulatory follicle competency

**DOI:** 10.1038/s41598-021-96473-7

**Published:** 2021-08-16

**Authors:** Sweta Ravisankar, Carol B. Hanna, Kelsey E. Brooks, Melinda J. Murphy, Nash Redmayne, Junghyun Ryu, Jason M. Kinchen, Shawn L. Chavez, Jon D. Hennebold

**Affiliations:** 1 Department of Cell, Developmental and Cancer Biology, Graduate Program in Molecular & Cellular Biosciences, Oregon Health & Science University School of Medicine, Portland, OR USA; 2 Division of Reproductive and Developmental Sciences, Oregon National Primate Research Center, Beaverton, OR USA; 3 Department of Obstetrics and Gynecology, Oregon Health & Science University School of Medicine, Portland, OR USA; 4 Metabolon, Inc., Morrisville, NC 27560 USA; 5 Department of Molecular and Medical Genetics, Oregon Health & Science University School of Medicine, Portland, OR USA

Correction to: *Scientific Reports* 10.1038/s41598-021-85704-6, published online 22 March 2021

Carol B. Hanna was omitted from the author list in the original version of this Article.

The Author Contributions section now reads:

“SR, CH, SLC, and JDH designed the study and performed experiments. SR, SLC, and JDH analyzed data, and wrote the manuscript. KEB and JR performed oocyte microinjections. NR assisted with oocyte collections and MJM helped perform the IHC experiments. JK helped with the metabolomics analysis.”

The original Article has been corrected.

