# Supplementary material for: Metabolomics analysis of follicular fluid coupled with oocyte aspiration reveals importance of glucocorticoids in primate periovulatory follicle competency
Source: Sci Rep. 2021 Mar 22;11:6506. doi: 10.1038/s41598-021-85704-6 (PMC7985310; doi:10.1038/s41598-021-85704-6)
Supplement: Supplementary file 1 — Supplementary Information 1. [file 41598_2021_85704_MOESM1_ESM.pdf]

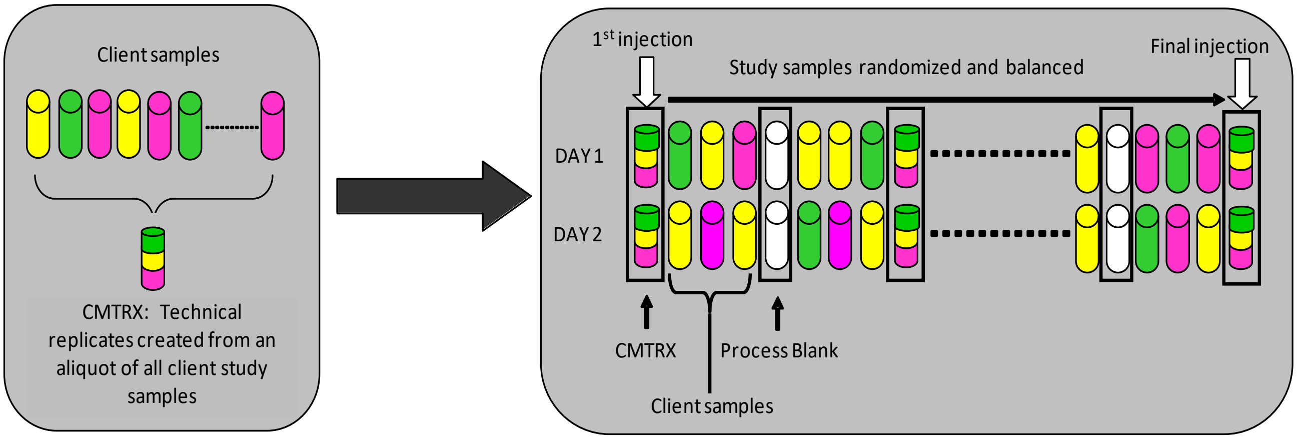

Supplementary Figure S1. Schematic representation of the pipeline used for metabolomics analysis. The process includes the preparation of technical replicates, quality control, randomization, and final injection for tandem MS analysis.
