# Supplementary material for: Metabolomics analysis of follicular fluid coupled with oocyte aspiration reveals importance of glucocorticoids in primate periovulatory follicle competency
Source: Sci Rep. 2021 Mar 22;11:6506. doi: 10.1038/s41598-021-85704-6 (PMC7985310; doi:10.1038/s41598-021-85704-6)
Supplement: Supplementary file 2 — Supplementary Information 2. [file 41598_2021_85704_MOESM2_ESM.pdf]

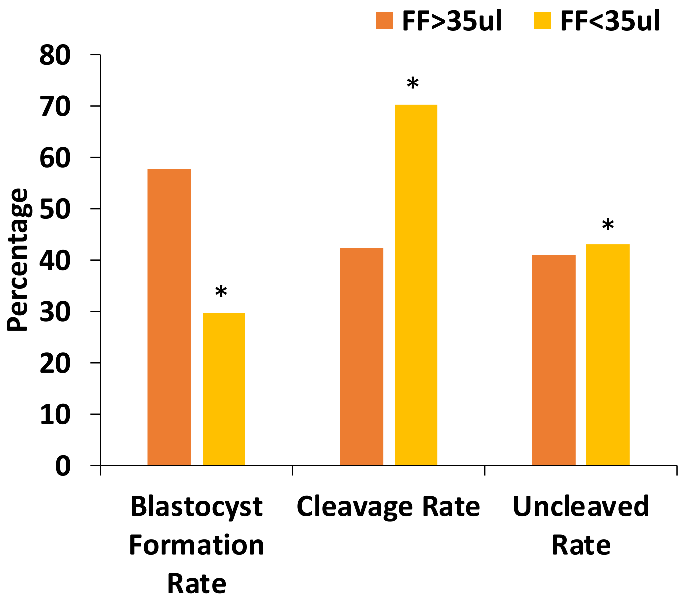

Supplementary Figure S2. The periovulatory FF volume impacts oocyte competency. A significant correlation was found by chi-square test (χ^2) between embryo groups, whereby a FF volume > 35 µl (orange bars) was associated with a higher likelihood of blastocyst formation, whereas a FF volume of < 35 µl (yellow bars) was associated with a greater probability of embryonic arrest before blastulation (χ^2=15.921, $p<0.001$).
