# Supplementary material for: Metabolomics analysis of follicular fluid coupled with oocyte aspiration reveals importance of glucocorticoids in primate periovulatory follicle competency
Source: Sci Rep. 2021 Mar 22;11:6506. doi: 10.1038/s41598-021-85704-6 (PMC7985310; doi:10.1038/s41598-021-85704-6)
Supplement: Supplementary file 3 — Supplementary Information 3. [file 41598_2021_85704_MOESM3_ESM.pdf]

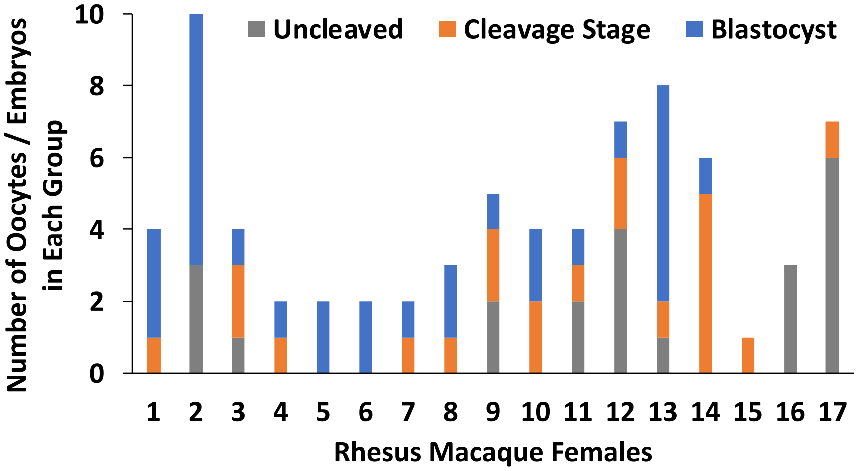


**Supplementary Figure S3.** Distribution of the female rhesus macaques from which all 74 FF samples were obtained. Each rhesus macaque female (N=17) is represented as a number on the x-axis and the number of corresponding oocytes and/or embryos in the uncleaved (gray), cleavage stage (orange), and blastocyst (blue) groups is shown on the y-axis.
