# Supplementary material for: Metabolomics analysis of follicular fluid coupled with oocyte aspiration reveals importance of glucocorticoids in primate periovulatory follicle competency
Source: Sci Rep. 2021 Mar 22;11:6506. doi: 10.1038/s41598-021-85704-6 (PMC7985310; doi:10.1038/s41598-021-85704-6)
Supplement: Supplementary file 4 — Supplementary Information 4. [file 41598_2021_85704_MOESM4_ESM.pdf]

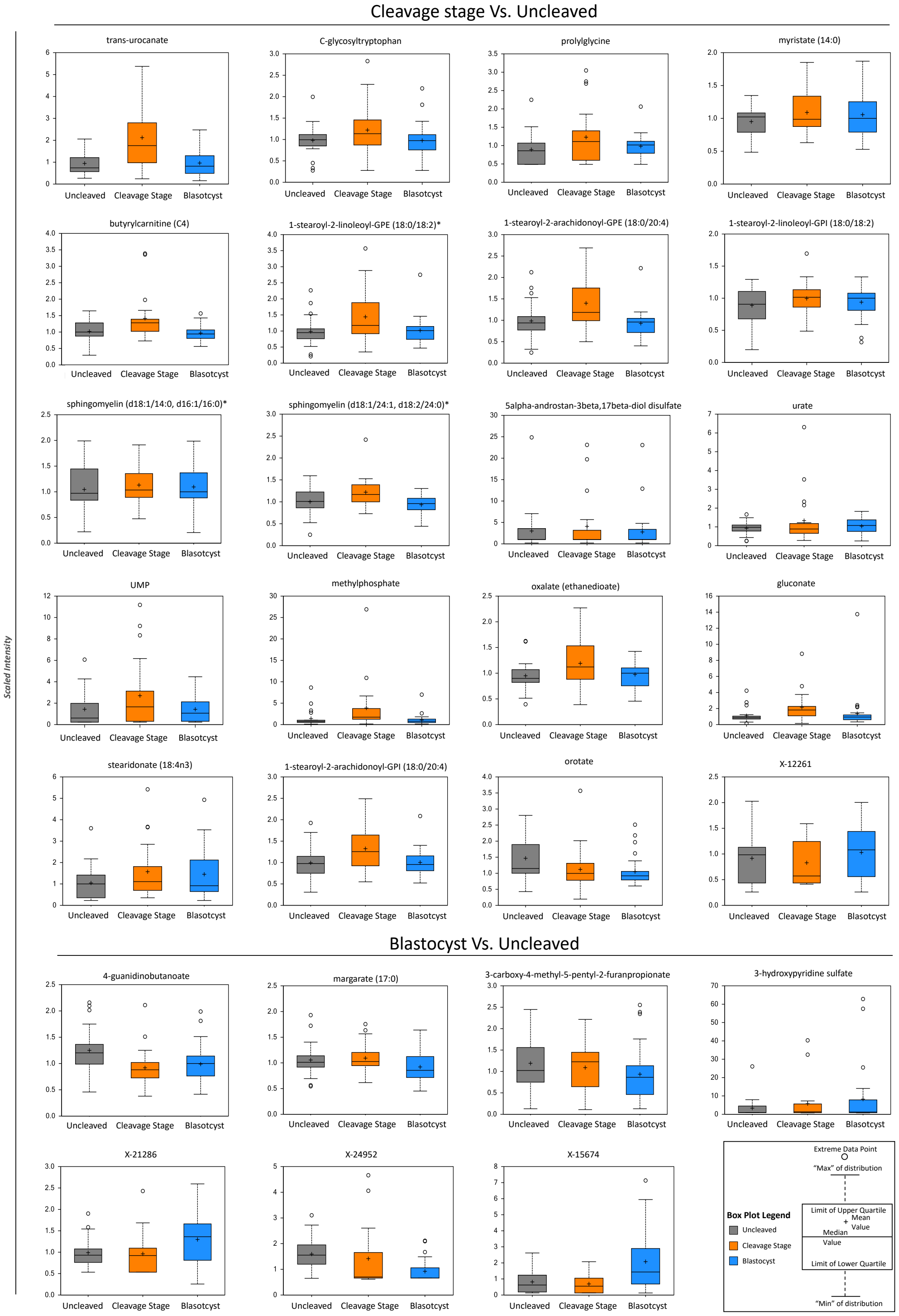

Supplementary Figure S4. Representative box plots of the statistically significant ($p<0.05$) metabolites between the FF samples belonging to the cleavage stage versus uncleaved groups as well as for the blastocyst versus uncleaved embryo groups.
