# Supplementary material for: Metabolomics analysis of follicular fluid coupled with oocyte aspiration reveals importance of glucocorticoids in primate periovulatory follicle competency
Source: Sci Rep. 2021 Mar 22;11:6506. doi: 10.1038/s41598-021-85704-6 (PMC7985310; doi:10.1038/s41598-021-85704-6)
Supplement: Supplementary file 5 — Supplementary Information 5. [file 41598_2021_85704_MOESM5_ESM.pdf]

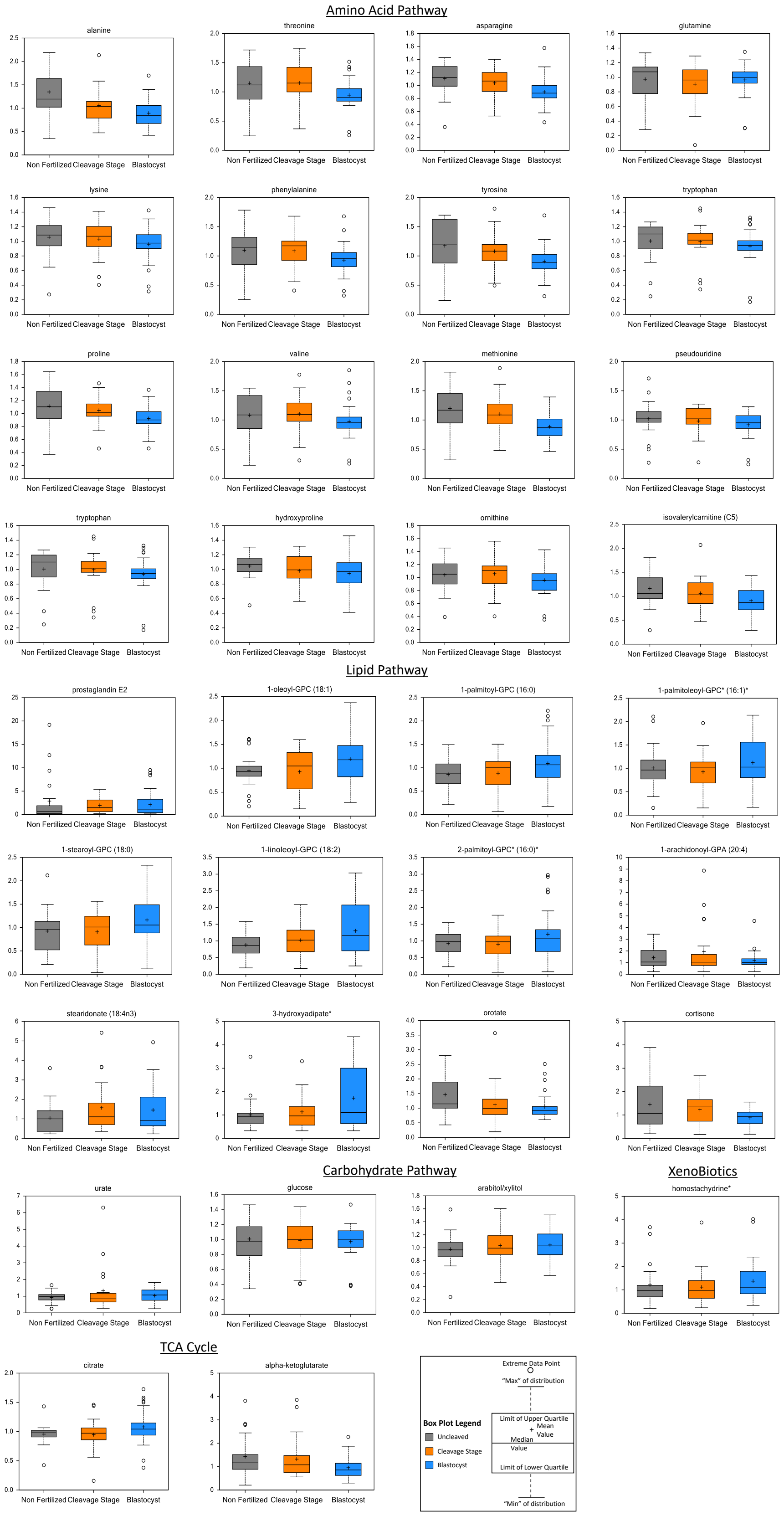

Supplementary Figure S5. Box plots of metabolites that exhibited a trend between the uncleaved, cleavage stage, and blastocyst embryo groups, but were not statistically significant.
