# Supplementary material for: Metabolomics analysis of follicular fluid coupled with oocyte aspiration reveals importance of glucocorticoids in primate periovulatory follicle competency
Source: Sci Rep. 2021 Mar 22;11:6506. doi: 10.1038/s41598-021-85704-6 (PMC7985310; doi:10.1038/s41598-021-85704-6)
Supplement: Supplementary file 6 — Supplementary Information 6. [file 41598_2021_85704_MOESM6_ESM.pdf]

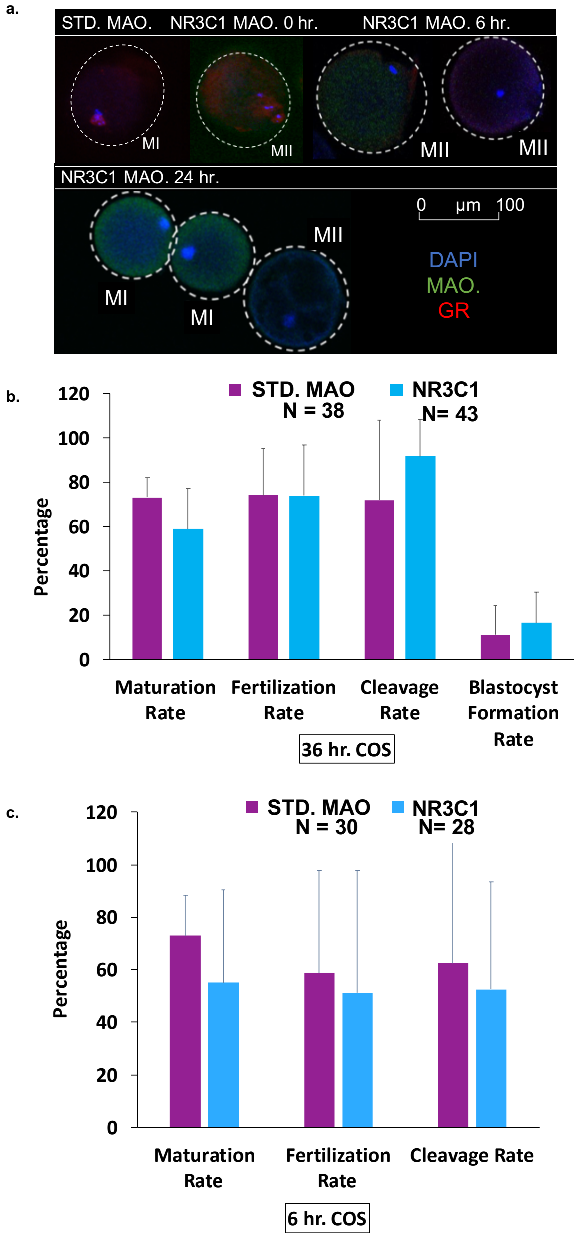

Supplementary Figure S6. NR3C1 knockdown in rhesus macaque oocytes has no effect on preimplantation development. **(a)** NR3C1 MAO knockdown was optimized in rhesus macaque oocytes obtained from a 36 h COS cycle and reduced expression confirmed between 0 h and 24 h post-microinjection using the STD MAO as a control. Both MAOs were tagged with 3'-carboxyflorescein (green) for visualization of successful microinjection and oocytes stained for DAPI (blue) and immunolabeled for NR3C1 (red). Note the decrease in the expression of NR3C1 in MI/MII oocytes at 6 h and 24 h after injection compared to 0 h and the STD MAO control. NR3C1 expression was reduced in rhesus macaque oocytes from **(b)** 36 h COS and **(c)** 6 h COS. Oocytes were fertilized 30 h post-hCG administration and allowed to undergo pre-implantation development post-IVF. The maturation, fertilization, cleavage, and/or blastocyst formation rates were calculated between STD. MAO (purple) and NR3C1 MAO (blue) injected oocytes and no differences were detected. N=number of oocytes microinjected with each MAO.
